# Superhydrophobic sand mulch and date palm biochar boost growth of *Moringa oleifera* in sandy soils via enhanced irrigation and nutrient use efficiency

**DOI:** 10.3389/fpls.2024.1434462

**Published:** 2024-11-26

**Authors:** Kennedy Odokonyero, Bob Vernooij, Batool Albar, Lisa Oki Exposito, Aishah Alsamdani, Amin Akhtar Ghulam Haider, Nayara Vivian Huve Musskopf, Najeh Kharbatia, Adair Gallo, Himanshu Mishra

**Affiliations:** ^1^ Environmental Science and Engineering (EnSE) Program, Biological and Environmental Science and Engineering Division, King Abdullah University of Science and Technology (KAUST), Thuwal, Saudi Arabia; ^2^ Water Desalination and Reuse Center (WDRC), King Abdullah University of Science and Technology (KAUST), Thuwal, Saudi Arabia; ^3^ Biological and Environmental Science and Engineering Division, Center for Desert Agriculture (CDA), King Abdullah University of Science and Technology (KAUST), Thuwal, Saudi Arabia; ^4^ Terraxy LLC, King Abdullah University of Science and Technology (KAUST), Thuwal, Saudi Arabia

**Keywords:** *Moringa oleifera*, evapotranspiration, metabolomic profiling, date palm biochar, superhydrophobic sand, soil amendments, transpiration, irrigation regimes

## Abstract

**Introduction:**

It is desirable to rehabilitate desert ecosystems with a selection of native plant species that render ecosystem services and yield natural products for creating a high-value industry, e.g., pharmaceuticals or cosmetics. However, plant growth under arid and hyper-arid conditions, such as in the Arabian Peninsula, is constrained by heat, freshwater scarcity, and alkaline sandy soils with low nutrient and water holding capacity. Therefore, it is imperative to develop nature-based sustainable technologies to improve arid soil conditions, as well as increase irrigation and nutrient-use eficiency.

**Methods:**

Here, we report on a study evaluating the effects of two complementary soil amendment technologies, namely Superhydrophobic sand (SHS) mulch and engineered biochar (EB) on the growth of *Moringa oleifera* plants. Effects of SHS (1cm-thick), EB (2% w/w), and SHS+EB treatments were tracked in greenhouse plants under normal (N, 100% field capacity) and reduced (R, 50% of N) irrigation scenarios for over 150 days, where EB treatments were pre-loaded with nutrients and remaining treatments received traditional NPK fertilizer.

**Results:**

Significant benefits of the SHS, EB, and SHS+EB treatments were found in terms of increased plant height, trunk diameter, leaf area, leaf chlorophyll content index, stomatal conductance, and shoot and root biomass in comparison with the controls. Evaporation water savings due to SHS mulching significantly enhanced transpiration under N and R scenarios. Similarly, EB and SHS+EB treated plants experienced higher transpiration than in the control plants under N and R conditions (*p*< 0.05). In response to water stress due to excessive evaporation, metabolomics analysis showed a higher accumulation of amino acids in control plants than other treatments under both irrigation regimes. Meanwhile, a higher abundance of sugars (i.e., D-Mannose, D-Fructose, glucose) and organic acid (i.e., malic acid) was observed in SHS and EB-treatments for Variable Importance in Projection (VIP) scores >1.0 (i.e., the scores considered of significance in contributing to the differences between treatment groups).

**Discussion:**

The results show the synergistic benefits of SHS and EB technologies for addressing the challenges of water scarcity and nutrient limitation in arid regions, which couldcontribute to the success and sustainability of agriculture and greening efforts in such regions.

## 1 Introduction

Climate change poses the greatest challenge to humanity’s future (IPCC, 2022), with its tremors already manifesting in terms of rising temperature, unpredictable precipitation and drought events, sea level rise, desertification, and increased aridity in dryland regions (IPCC, 2023; Berdugo et al., 2020; FAO, 2011; Berdugo et al., 2019). As a result, it is crucial to realize giga-scale carbon drawdown via reducing emissions and long-term carbon capture. In this context, arid lands present tremendous challenges and opportunities. Spanning over ~40% of the land surface, they house over 2 billion people and are characterized by degraded soils, perils of organic waste landfilling and carbon emissions (Berdugo et al., 2020), and a substantial desire for ecosystem services and regional food security. In response, several nations have embarked upon massive greening projects, especially in the Middle East and Northern Africa (MENA) and Asia, including China’s Kubuqi Ecological Restoration (Lyu et al., 2020), the Bamberger Ranch Preserve in Texas–USA (Greene, 2007), and the Saudi and Middle East Green Initiatives (Hong et al., 2023). Growing plants in arid and hyper-arid climates, with extremely low annual precipitation and high evapotranspiration, renders their survival difficult (Sultana and Nasrollahi, 2018; Sahour et al., 2020). Therefore, it would be prudent to recruit native plants, which have evolved adaptations to survive under abiotic stresses due to heat, drought, nutrient limitation, and salinity (Zandalinas et al., 2018; Jenks and Hasegawa, 2005; Khalik et al., 2013), and utilize treated wastewater and sustainable soil amendment technologies to establish them (Hong et al., 2023). Sustainability of these projects could be furthered if the plants also contribute valuable natural products for high value industry, e.g., pharmaceutical or cosmetics, thereby contributing to job creation, industrial development, and economic prosperity.

A popular drought-tolerant plant native to the MENA region is *Moringa* (spp. *M. peregrina* or *M. oleifera*), a perennial tropical deciduous tree of Moringaceae family with economic, ornamental and pharmacological value proposition (Gao et al., 2020; Cao et al., 2023; Senthilkumar et al., 2018). Recently, Saudi Arabia’s Al-Ula Governorate signed a joint agreement with the *Dior Company* towards planting millions of *M. peregrina* trees to harness their potential in creating a high-value perfume and cosmetic industry (Arab-News, 2022). Although *M. oleifera* can survive under low soil nutrients and irrigation conditions (Alegbeleye, 2018), we wondered how its establishment may vary with the application of two soil amendments developed in our group, namely Superhydrophobic Sand (SHS) mulch and Engineered Biochar (EB). SHS is a biomimetic super-water-repellent material comprising of sand grains coated with a nanoscale layer of biodegradable paraffin wax (Mishra et al., 2022). Its application as a 0.5–1 cm-thick mulch layer on moist soil curtails the evaporative water loss, which promotes plant health (Gallo Jr et al., 2022; Odokonyero et al., 2022). Unlike plastic mulch, SHS degrades in the soil over time due to microbial activity, obviating the need of landfilling (Kasirajan and Ngouajio, 2012; Barnes et al., 2009). The second technology (EB) is derived from the pyrolysis of date palm leaves residues in the range of 500–600°C range. We chose date palm because it contributes to over 200,000 tons of organic waste in KSA annually (Faiad et al., 2022); as this waste is landfilled or burned, it releases greenhouse gases into the air. In contrast, during pyrolysis, the biomass is transformed into a stable form of carbon that persists in the soil for 100s for years. EB fashions high cation-exchange capacity, which sandy soils lack, therefore its addition to sandy soil enhances the soil’s nutrient and water retention capacity, improves soil health, and promotes plant growth (Joseph et al., 2021; Bolan et al., 2022; Lehmann and Joseph, 2009; Van Zwieten et al., 2010). Thus, the deployment of SHS and EB could stimulate arid land revegetation with crops, native trees, shrubs and pastures via enhanced soil moisture and nutrient content, which promote transpiration and photosynthesis (Caldera and Breyer, 2023; Lefebvre et al., 2019; Farzi et al., 2017; Zhang et al., 2018).

Here, we fill this knowledge gap by quantifying the stand-alone and synergistic effects of SHS and EB on the growth of *M. oleifera* under normal and reduced irrigation conditions for 21 weeks. To probe the efficacy of EB on the nutrient-use efficiency and identifying new best practices, we pre-loaded nutrients onto EB and then did not provide fertilizer supplementation to the EB-treated or EB+SHS-treated plants, while the control and the SHS-treated plants received weekly nutrient dosing (See Methods for details). We hypothesized that SHS would reduce evaporation and enhance water use efficiency while, the EB would enhance nutrient use efficiency during plant establishment, ultimately improving plant morpho-physiological and metabolomic responses.

## 2 Materials and methods

### 2.1 Superhydrophobic sand and engineered biochar

SHS and EB were procured from Terraxy LLC (Saudi Corporate License: 4030502411). SHS production entailed mixing heated silica sand with molten paraffin wax in a 1000:1 mass ratio (Mishra et al., 2022; Gallo Jr et al., 2022; Odokonyero et al., 2022). The wetting properties of the SHS were characterized via contact angle measurements and scanning electron microscopy (SEM), following a previous protocol (Gallo Jr et al., 2022). The materials production process for EB was as follows: First, date palm leaves were collected from KAUST Horticultural Department (Makkah, Saudi Arabia) and dried under the sun. The dried biomass was then tightly packed inside a batch reactor and pyrolyzed in the absence of oxygen at the temperature of > 500°C. The resulting biochar was crushed and ground into smaller particles, followed by loading with macronutrients (diammonium phosphate) and micronutrients (Ca, Mg, S, Fe, Mn, Zn, Cu, B, and Mo) to realize the EB (See **Table 1** for elemental composition). Thereafter, the cationic exchange capacity (CEC) of the EB was determined by an adapted Mehlich-3 protocol for base cations (Ziadi and Tran, 2007). Briefly, the EB was first thoroughly washed until the EC of the water was< 0.2 mS/cm. Then, 0.5 g of EB was mixed with 1:10 m/v Mehlich-3 solution and placed in the shaker at 200 rpm for 3-4 hours. The mixture was then filtered and the base cations (Ca2+, Mg2+, K+, Na+) concentrations was measured through Inductively Coupled Plasma Optical Emission Spectroscopy, ICP-OES (Agilent Technologies 5110). The CEC from the base cations was added and divided by the biochar mass to get CEC in cmol/kg, considering the charges of the cations. Prior to scanning electron microscopy (Zeiss Merlin SEM, Carl Zeiss SMT AG), EB was adhered to the stub via carbon tape and coated with a 1nm iridium layer.

**Table 1:** Soil chemical properties before and after the experiment, i.e., *t* = 0 and 21 weeks.

	Soil pH, EC, Salinity			Elemental concentrations (ppm)											Inorganic Nitrogen		
Treatments	pH (1:2, w/v)	EC (µS/cm)	Salt (ppm)	Al	Ca	Cu	Fe	K	Mg	Mn	Na	P	S	Zn	NH_4_–N (mg/kg)	NO_3_–N (mg/kg)	Total inorganic N (mg/kg)
**Control (*t_0_*)** **EB, no soil (*t_0_*)** **Control-N (After)**	**7.9** **8.5** **7.7**	**420** **-** **2631**	**-** **-** **3368**	**624** **-** **578**	**3237** **6582** **3410**	**1.25** **18.1** **9.77**	**286** **300** **248**	**220** **1063** **812**	**617** **1549** **679**	**39.1** **64.7** **45.6**	**414** **1267** **758**	**42.2** **5641** **142**	**51.2** **568** **80.8**	**2.32** **16.8** **12.9**	**0.37 ± 0.02** **-** **0.52 ± 0.19**	**16.2 ± 0.91** - **7.61 ± 0.70**	**16.57 ± 0.46** **-** **7.61 ± 0.70 8.13 ± 0.44**
**SHS-N (After)**	**7.5**	**769**	**986**	**535**	**2870**	**2.81**	**214**	**726**	**560**	**43.3**	**596**	**232**	**44.4**	**17.2**	**0.54 ± 0.13**	**4.41 ± 0.82**	**4.95 ± 0.47**
**EB-N (After)**	**8.4**	**679**	**869**	**486**	**3104**	**3.52**	**188**	**357**	**567**	**44.7**	**733**	**230**	**54.6**	**15.8**	**0.23 ± 0.04**	**2.42 ± 0.59**	**2.65 ± 0.31**
**SHS+EB-N (After)**	**8.8**	**157**	**201**	**456**	**2381**	**2.50**	**196**	**206**	**483**	**36.0**	**518**	**187**	**42.3**	**12.4**	**0.25 ± 0.02**	**0.41 ± 0.06 0.66 ± 0.04**	**0.41 ± 0.06**
**Control-R (After)**	**7.8**	**1792**	**2294**	**283**	**1449**	**0.95**	**126**	**280**	**297**	**20.1**	**312**	**39.1**	**28.6**	**6.23**	**0.92 ± 0.35**	**7.17 ± 0.31**	**8.09 ± 0.33**
**SHS-R (After)**	**8.4**	**697**	**892**	**587**	**3080**	**1.57**	**247**	**346**	**593**	**42.2**	**622**	**63.3**	**56.9**	**12.9**	**0.55 ± 0.38**	**2.45 ± 0.40**	**3.00 ± 0.39**
**EB-R (After)**	**8.4**	**596**	**763**	**581**	**2991**	**3.93**	**246**	**383**	**647**	**50.1**	**699**	**224**	**62.2**	**14.5**	**0.20 ± 0.05**	**2.36 ± 0.11**	**2.56 ± 0.08**
**SHS+EB-R (After)**	**8.6**	**148**	**189**	**501**	**2830**	**3.27**	**217**	**252**	**537**	**40.4**	**535**	**215**	**50.3**	**12.4**	**0.26 ± 0.02**	**1.02 ± 0.67**	**1.28 ± 0.34**

Changes in the soil pH, electrical conductivity (EC), salinity, elemental and inorganic nitrogen (NH_4_–N and NO_3_–N) concentrations in the control soil, the SHS-treated soil, the EB-treated soil, and the SHS+EB-treated soil under normal (**N**) and reduced (**R**) irrigation. Values presented are means from 5 replicates (*n* = 5) and standard error (±) values given for inorganic N content. “Control (*t_0_*)” and “EB, no soil (*t_0_*)” refer to the control soil and engineered biochar before the experiment, respectively. During the experiment, EB was mixed with soil at a 2% concentration by mass.

### 2.2 Plant growth conditions, treatments and experimental designs

A controlled greenhouse experiment was conducted using *M. oleifera* plants at the Plant Growth Core Labs, King Abdullah University of Science and Technology, Saudi Arabia. Seeds of *M. oleifera* were sown in plastic trays using potting mix from Stender AG (Schermbeck, Germany) and grown for five weeks before transplanting. Prior to watering, bottom-sealed plastic pots were filled with about 4 kg dry sandy soil (See **Table 1** for elemental composition); the soil in each pot for EB treatment was uniformly mixed with 2% of EB (i.e., 80 g dry mass per pot). After EB application, the seedlings were transplanted, and pots were watered to two field capacity: pots under 100% field capacity for normal (N) irrigation and those under 50% field capacity for reduced (R) irrigation. After irrigation, 1cm-thick layer of SHS mulch was applied on top of the pots separated for SHS mulch treatment. The initial weight of each pot at their respective irrigation regime was gravimetrically determined to track for water loss on a weekly basis. A total of 64 pots were prepared and irrigated: 40 pots with plants and 24 pots without plants. Pots without plants were used to quantify water loss due to soil evaporation (E) only while those with plants were used for gravimetric quantification of evapotranspiration (ET). The EB used for this experiment was pre-loaded with nutrients and did not receive further fertilizer application, while the control and SHS treatments received fertilizers at a dose of 200 ml/week of 2 g/L of NPK (20-20-20) solution. The treatment combinations used in this study are as follows: Control-N, Control-R, SHS-N, SHS-R, EB-N, EB-R, SHS+EB-N, and SHS+EB-R; each with five replicates including pots without plants. All pots were completely randomized giving a 2 × 2 factorial design involving two experimental factors of soil amendment treatment and irrigation regime, each with two levels. Plants were grown in the greenhouse for 21 weeks at 28/19 ± 2°C (day/night), ~800 µmolm^-2^ s^-1^ photosynthetically active radiation, and 45-60% ± 5% relative humidity.

### 2.3 Evapotranspiration

We partitioned ET into soil evaporation and transpiration by performing gravimetric measurements of pot weight on a weekly basis until harvest date. We monitored water loss in pots and adjusted water levels by weighing each pot and adding corresponding amount of water to compensate for the water lost. Weekly ET was taken as total water lost from each pot with plants between the day of irrigation and the next date of weighing the pots in the following week using:


(1) ET=(Initial weight of pot with plant-final weight of pot with plant)

#### 2.3.1 Evaporation

By assuming that evaporation from pots without plant equals to evaporation from pots with plants, we estimated weekly water loss by evaporation using pots without plants from the expression:


(2) Evaporation=(Initial weight of pot without plant-final weight of pot without plant)

#### 2.3.2 Transpiration

Weekly transpiration was determined from the expression:


(3) Transpiration =(weekly ET in each pot-weekly evaporation)

### 2.4 Plant parameters

#### 2.4.1 Plant height

We measured plant height at the beginning of the experiment (i.e., at transplanting) followed by bi-weekly measurements. We monitored changes in plant height over time and calculated the mean plant height at the end of the experiment for each treatment.

#### 2.4.2 Leaf area

To determine plant leaf area, we took photos from each plant after every two weeks and processed the images. Leaf canopy images were processed using Easy Leaf Area Free software (https://www.quantitative-plant.org/software/easy-leaf-area). We used the RGB value (relative area) of each pixel (i.e., 2 × 2 cm red paper) to identify the leaf and scale regions in each image. Based on this pixel area, we calculated the total area of leaves (canopy size) for each plant. As the RGB scale for green areas remained, the background colors were removed.

#### 2.4.3 Trunk diameter

After every two weeks, we measured the trunk diameter of each plant using a digital engineer caliper, to track the increase in girth. We conducted measurements of the trunk around the lower trunk (base height).

#### 2.4.4 Stomatal conductance and leaf chlorophyll content

We determined leaf stomatal conductance using AP4 Porometer (Delta T, Cambridge, UK). We performed measurements on three young but fully expanded leaves once a week (between 10:00 AM–12:00 noon) and calculated the mean conductance for each treatment combination. Leaf chlorophyll content index, (CCI) was measured weekly using CCM-200 Chlorophyll Content Meter (Optic-Sciences, Inc. Hudson NH03051, USA), with measurements being performed on four young but fully expanded leaves.

#### 2.4.5 Plant biomass

At harvest, we separated the plants into shoot and root components and determined their fresh mass. We then removed leaf samples from each plant for metabolomics profiling and stored the samples at -80°C until the time for analysis. We put the other shoot and root samples in paper bags and dried them in an oven at 105°C for 72 hours after which, their dry mass was determined.

### 2.5 Metabolomics analysis and data processing

For metabolomics analysis, stored leaf samples in vials were immersed in liquid nitrogen and crushed repeatedly using microbeads in the Geno/Grinder^®^ machine at 1200 rpm for 1 min. and the leaves were frozen in liquid nitrogen and lyophilized to dryness. Cells were later busted using the bead blaster at 4000 rpm for 5×1:30 minutes at -10°C. For metabolites extraction, 25mg of each sample was transferred to an Eppendorf vial and 1000 µL of 3:1 Methyl-tert-butyl/methanol, followed by shaking at 4°C for 45 minutes and sonicating in an ice bath for 20 minutes. Subsequently, we added 650 µL of 3:1 methanol/water mix with shaking for 1 minute, followed by centrifuging the samples for 5 minutes at 10000 rcf to achieve the phase separation. We then transferred the top organic (MTBE) layer to GC-vials for direct injection in order to test for hydrocarbon contents, while the bottom aqueous layer was transferred to another vial where subjected to complete dryness using Centrivap. After drying, we added 30 µL of MOX reagent, followed by rigorous mixing for 1 minute and incubation at 37°C for 90 minutes at 600 rpm. Subsequently, we added 50 µL of the trimethylsilylation (TMS) derivatization reagent i.e., N,Obis(trimethylsilyl)trifluoroacetamide (BSTFA), followed by another incubation at 30°C for 45 minutes at 600 rpm.

After sample preparation, we then performed a non-targeted primary metabolites analysis using a single quadrupole GC-MS (Agilent 7890 GC/5975C MSD) operated under electron ionization (EI) at 70 eV. We analyzed each derivatized sample using both split (20:1) and splitless direct injection into the GCMS inlet. A DB-5MS fused silica capillary column (30 m × 0.25 mm I.D., 0.25 µm film thickness; Agilent J&W Scientific, Folsom, CA) was utilized for chromatographic separation, which was chemically coupled with a 5% phenyl methylpolysiloxane cross-linked stationary phase. Helium was utilized as the carrier gas at a constant flow rate of 1.0 mL min^-1.^ Metabolites were separated using an oven gradient temperature. The initial oven temperature was set to 70°C for 4 min, then ramped at a rate of 6°C/min to 330°C with a 5-min hold time. The GC inlet temperature was set at 250°C, and the temperature of the transfer line to the MS EI source was kept at 320°C. The MTBE layer was analyzed through direct split injection, looking for differences in hydrocarbon levels, but we found no significant differences.

We processed the GC–MS data using MS-Dial (version 4.9.22121) using a combined MSP library of NIST2020 and GMD (Golm metabolomics Database), freely available online. We performed splitless injection, but due to oversaturation of the sugar region, data regarding peaks with a retention time between 28 and 34 was based on a 1:20 split injection of the same samples. For both, the mass scan range was set at 35-700 Da, with a retention time range of 9–50 min. Minimum peak height was set at 10000. Default deconvolution parameters and identification settings were used. Peak count filter was set at 12.5%, with an N% minimum of 60% per group. Blanks were used to remove background noise and pooled samples were used for alignment in combination with the retention index of an alkane standard. Peak validation was performed in Agilent MSD Productivity ChemStation. Statistical analysis was performed in MetaboAnalyst, comprising the analysis of Variable Importance in Projection (VIP) and Partial Least Square Discriminant Analysis (PLS-DA) scores after normalization by sum and auto-scaling.

### 2.6 Soil sample analysis

At the time of harvest, we collected soil samples from each pot and oven-dried them at 35°C for 7 days before performing soil chemical analysis. To analyze Ca, Mg, Na, K, P, S, Fe, Mn, Cu, and Zn, we weighed 0.5 g of soil sample and extracted using 10 mL of Mehlich 3 solvent (Zbíral, 2000; Tran and Ziadi, 2007). The mixture was shaken for 4 hours, filtered, and the liquid component was analyzed using Agilent Inductively Coupled Plasma Optical Emission Spectroscopy (ICP-OES). For inorganic nitrogen (ammonium, NH_4_−N and nitrate, NO_3_−N) analysis, 1 g of soil sample was weighed and extracted using 10 mL of 1M KCl, while shaking for 1 hour. The solution was filtered, and the liquid was analyzed using the Salicylate Method for low range nitrogen ammonia (Giner-Sanz et al., 2020) (HACH kit no. 2604545) and the 2.6-Dimethylphenol method for nitrogen nitrate (Moorcroft et al., 2001) (HACH kit no. LCK 339). To analyze pH and TDS (salinity), 5 g of the sample was mixed with 10 mL of water for 1 hour. The pH and electrical conductivity (EC) of the resulting slurry (w/v of 1:2) were measured using a Seven Compact Duo with InLab probes. See **Table 1** for the soil analysis of all the treatments at the beginning and end of this study.

### 2.7 Data analysis

The plant data collected for ET, growth and biomass was analyzed using Origin Pro software (Version 2021). We used a two-way analysis of variance (ANOVA) to analyze the effects of soil amendment and irrigation regime on the variables measured. We subjected all data collected to normality tests; all data conformed to normal distribution requirement. We used Tukey test for 0.05 level of statistical significance to perform multiple comparison of treatment means.

## 3 Results

### 3.1 Superhydrophobic sand and engineered biochar

**Figure 1:**
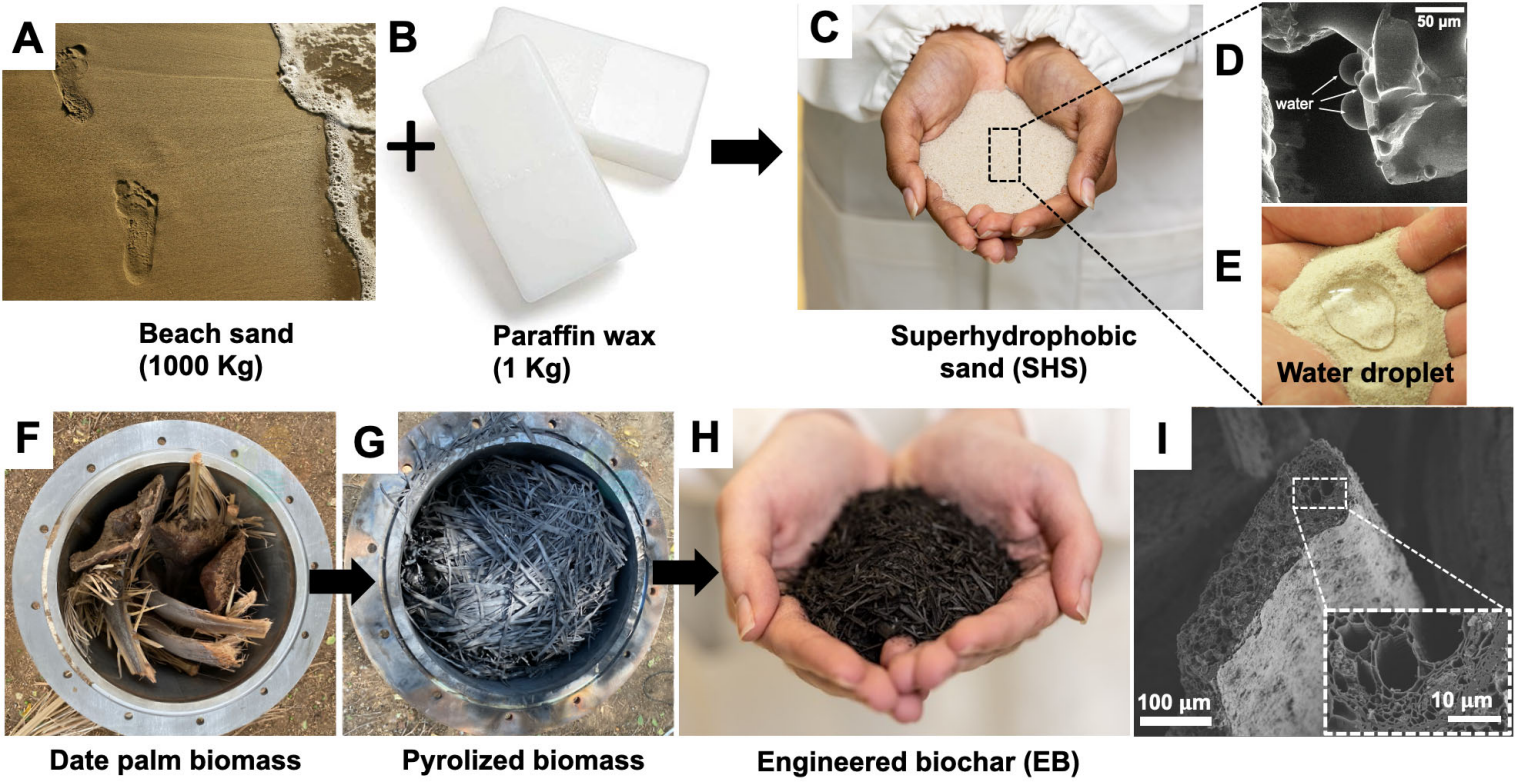
Photographs showing the production and characterization of superhydrophobic sand (SHS) and engineered biochar (EB). **(A–C)** thermal combination of silica sands and paraffin wax (1000:1 ratio) to produce SHS with conformal coating of sand grains using thin layer of wax; **(D, E)** scanning electron microscope (SEM) and macroscale images demonstrating the water-repellent (superhydrophobic) behavior of the SHS by moisture droplets on sand grains. **(F, G)** date palm biomass converted to biochar using a batch pyrolysis reactor at > 500°C; **(H)** post-processed biochar modified into EB by grinding to smaller particles followed by nutrient enrichment (i.e., loading with macro- and micronutrients). **(I)** SEM images of EB at 100 μm and 10 μm (Inset) showing the highly porous structure of the biochar alongside xylem and tracheids. Note: Panels **(D, E)** were adapted from our previous work: (Gallo Jr et al., 2022).

During SHS preparation, as heated silica sand was mixed with molten paraffin wax, a conformal coating of sand grains with wax was realized (**Figures 1A–C**). SEM imaging revealed grain-level hydrophobic behavior of SHS due to its paraffin coating (**Figure 1D**). At the macroscale, SHS exhibited superhydrophobicity due to the entrapment of air at the water–solid interface (**Figure 1E**), characterized by apparent contact advancing and receding angles of *θ*_A_ ≈ 160° and *θ*_R_ ≈ 150°, respectively for water droplets of 10 µL volume advanced and retracted at the rate of 0.2 µL/s (Gallo Jr et al., 2022).

The EB production entailed adding date palm leaves to a barrel with a concentric outer drum with heat supplied by burning liquified petroleum gas. The inner barrel temperature fluctuated in the range of 500–650°C (**Figures 1F, G**). The resulting raw biochar was then post-processed by Terraxy, following its proprietary protocols, to realize EB with nutrient-loading and the pH 8–8.5 (**Figure 1H**, see Methods, **Table 1**). The CEC of the EB was found to range between 37–40 cmol/kg. Surface characterization of the EB using SEM demonstrated significant degree of porosity within the biochar matrix (**Figure 1I**).

### 3.2 Plant growth

**Figure 2:**
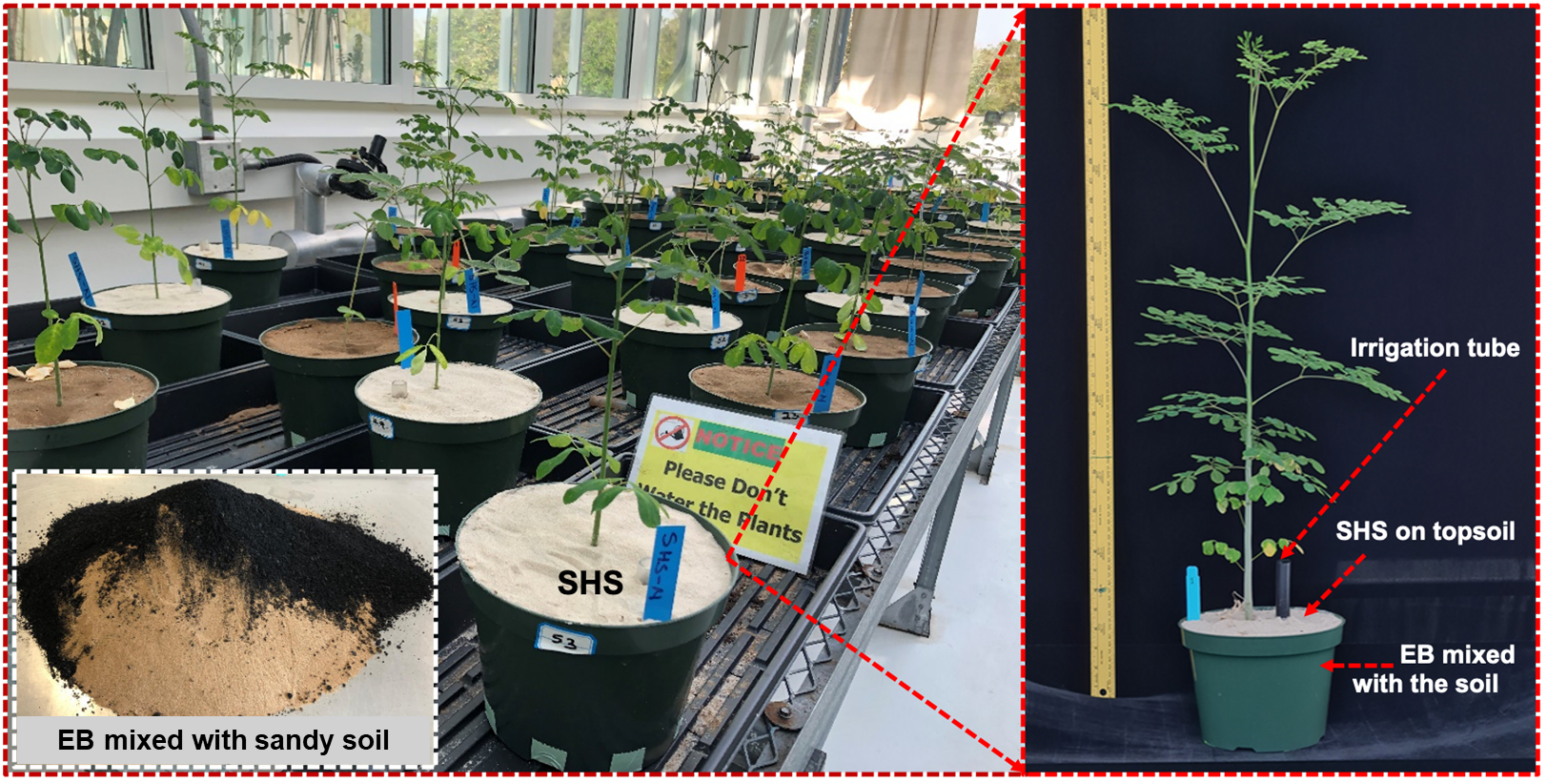
Potted *Moringa oleifera* plants in the greenhouse grown using control soil, Superhydrophobic sand (SHS), Engineered biochar (EB), and their combination (SHS+EB). The EB was mixed with the sandy soil (inset) and pots were filled with the soil under each treatment. White silica sand layer on top of the pots is for either the SHS mulch or SHS+EB treatments; control pots represent the pots without SHS or EB treatment.

When EB was homogeneously mixed with the regional sandy soil (**Figure 2**–inset) and filled in the respective pots for subsequent irrigation and seedling transplanting, we ensured no downward percolation of water by sealing off the holes at the bottom of the pots. SHS was applied on top of the moist soil as a 1 cm-thick layer, and irrigation was applied through a tube to the soil underneath. **Figure 2** illustrates the experimental setup from soil preparation to plant establishment. Over the growing period, the morphological attributes of the plants from different soil amendment treatments were tracked and compared. During this time, we also tracked the changes in pot mass every week – i.e., pots with and without plants – which enabled us to pinpoint the contributions of evaporation (E) and transpiration (T).

#### 3.2.1 Plant height and trunk diameter

**Figure 3:**
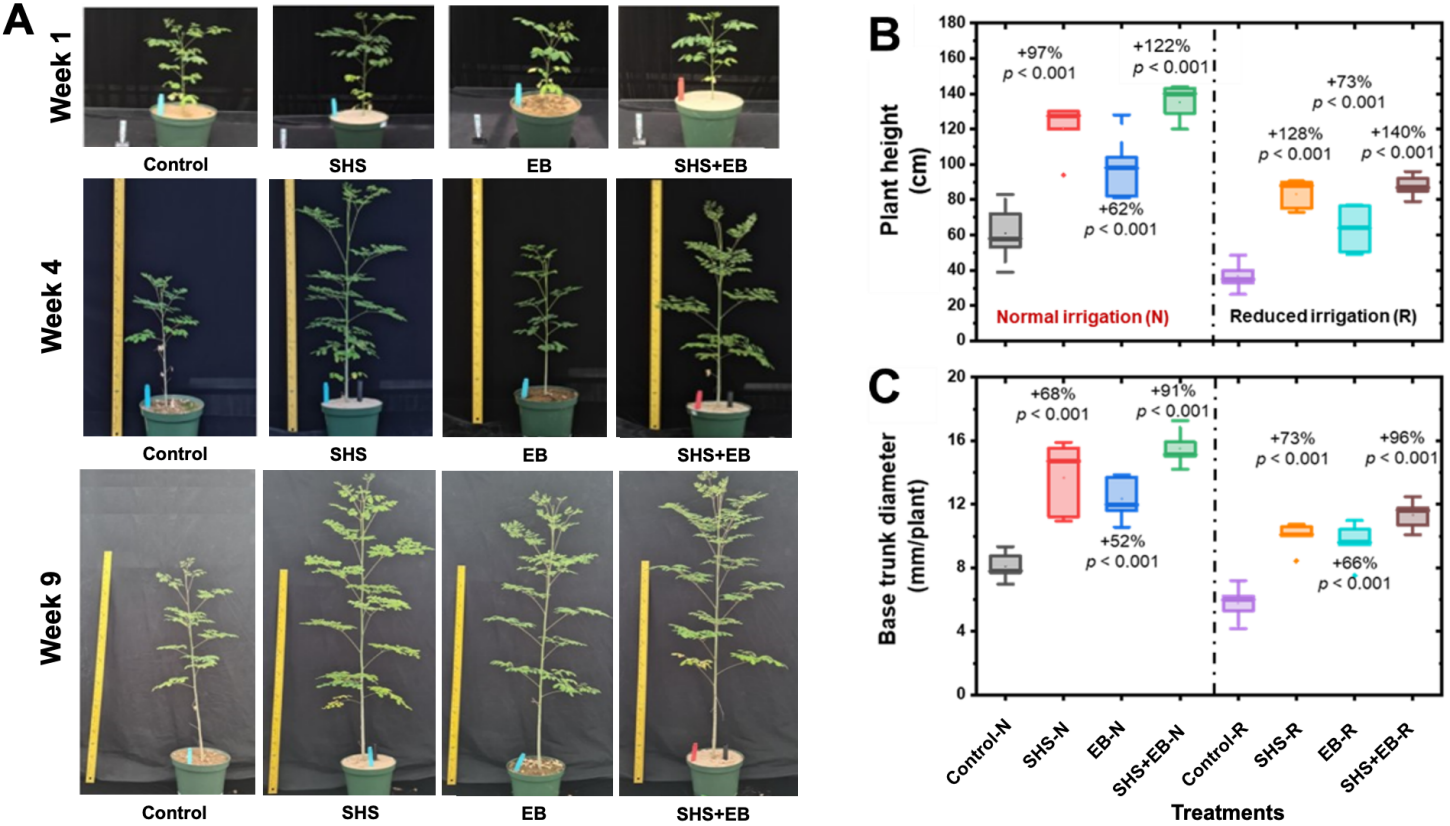
**(A)** Representative snapshots of potted Moringa oleifera plants in the greenhouse under normal (N) irrigation grown using the following treatments: Control, SHS mulch, EB, and SHS+EB. Boxplots showing: **(B)** mean final plant height at week#21; **(C)** Mean base trunk diameter for plants under N and R irrigation scenarios at 21 weeks after transplanting. Each box represents the data distribution from 5 replicates (n = 5; N=40) with the mid-line indicating the median value, the dot inside the box represents the mean value, the upper and lower sections of the box represent the 25% and 75% of data points, respectively, and the whiskers on the box represent the 1.5 interquartile range, the dot outside the box indicates outlier. Percentage differences between treatments are presented relative to control (bare) soil along with their corresponding p-values derived from two- way ANOVA using p< 0.05 level of statistical significance.

The growth of *M. oleifera* plants followed this order as a function of treatments: Control< EB< SHS< SHS+EB. The plants grew taller and bigger in size throughout the course of their life as investigated here (**Figure 3A**). Under N irrigation, mean plant height significantly increased in SHS, EB and SHS+EB plants by 97%, 62%, and 122%, respectively relative to the controls (**Figure 3B**). Under R irrigation, mean plant height was higher in SHS, EB, and SHS+EB than their control counterparts by 128%, 73%, and 140%, respectively. The base trunk diameter also increased significantly due to SHS, EB, and SHS+EB treatments by 68%, 52%, and 91% when N irrigation was used (**Figure 3C**). Following this trend, base trunk diameter under R irrigation increased by 73%, 66%, and 96% for SHS, EB, and SHS+EB treatments in comparison with the control plants (p< 0.05).

#### 3.2.2 Leaf area

**Figure 4:**
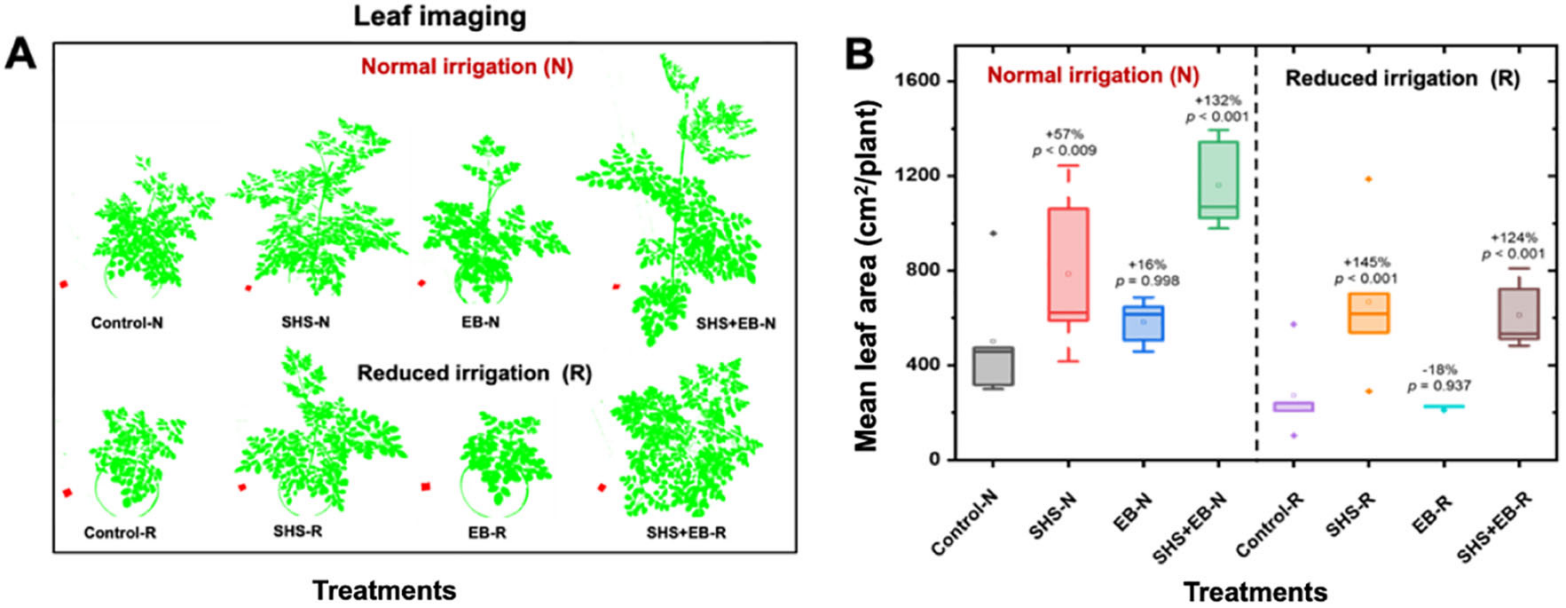
Measurement of leaf area. Processed images of plant leaf canopies **(A)** and mean leaf area **(B)** of *Moringa oleifera* plants grown in control, superhydrophobic sand (SHS), engineered biochar (EB) and their combination (SHS+EB) under normal (N) and reduced (R) irrigation. Leaf canopy images were processed using Easy Leaf Area Free Software (https://www.quantitative-plant.org/software/easy-leaf-area). We used the RGB value (relative area) of each pixel (i.e., 2×2 cm red paper) to identify the leaf and scale regions in each image. As the RGB scale for green areas remained, the background colors were discriminated.

From the visual depiction in **Figure 4A**, the canopy sizes for plants followed the order: Control ≈ EB< SHS< SHS+EB. Leaf area for SHS and SHS+EB treatments were significantly larger than in control treatment under N irrigation by 57% and 132%, respectively; and by 145% and 124%, respectively under R irrigation (**Figure 4B**). When changes in leaf area were followed between week 11 and week 15 of plant growth, mean leaf area significantly increased over time in the order: SHS+EB > SHS > EB > control (See **Supplementary Figure 1**).

### 3.3 Evapotranspiration partitioning

#### 3.3.1 Cumulative ET over time

**Figure 5:**
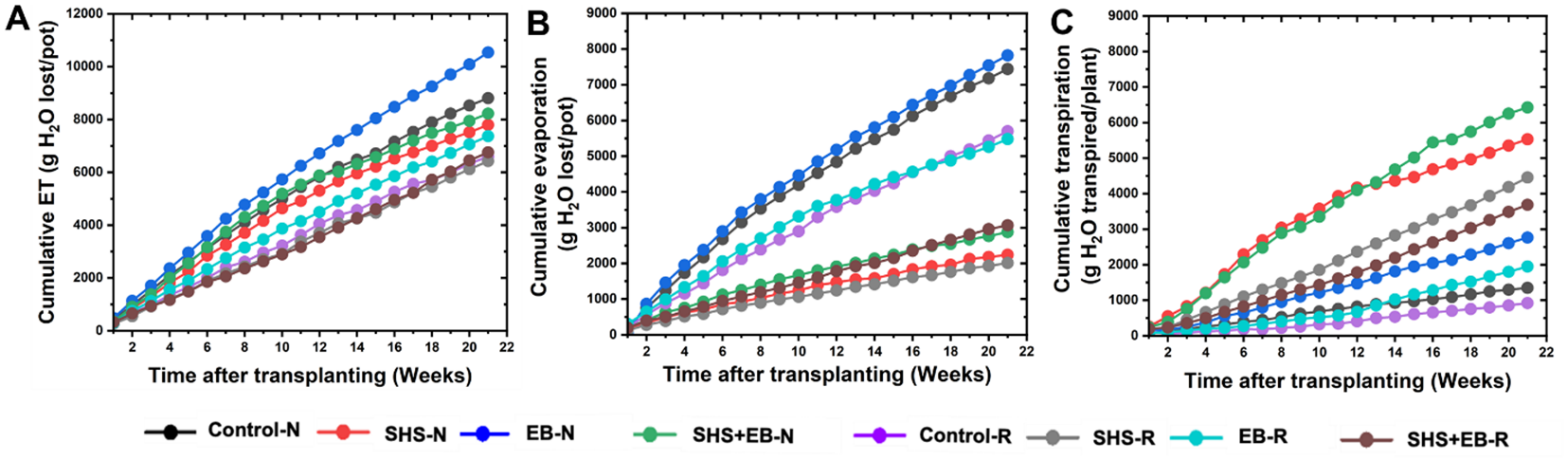
Partitioning of cumulative ET **(A)** into cumulative evaporation **(B)** and cumulative transpiration **(C)** during 21 weeks for *Moringa oleifera* plants grown in control soil, Superhydrophobic sand (SHS), engineered biochar (EB) and their combination (SHS+EB) under normal (N) and reduced irrigation (R) during a period of 21 weeks after transplanting. Each data point is a mean of five replicates (*n* = 5).

Throughout the growing period, “cumulative” ET was the highest in the EB treatment followed by the control (**Figure 5A**), while evaporation across growth period was higher in the EB and the control cases than in the rest of the treatments. For the SHS-treated plants, cumulative evaporation under N and R irrigation was only 28% and 31% of the total ET, respectively, leaving ~70% soil moisture for transpiration (**Figures 5B, C**). In the EB treatment, cumulative evaporation under either N or R irrigation was 74% of the cumulative ET, leaving a tiny 26% for transpiration. For the SHS+EB treatment, cumulative evaporation was 35.5% and 44.8% of the total ET under N and R respectively. As expected, cumulative evaporation in the controls was the highest, i.e., up to 84.7% and 86% of the total ET under N and R irrigation, respectively.

#### 3.3.2 Total ET and normalized transpiration

**Figure 6:**
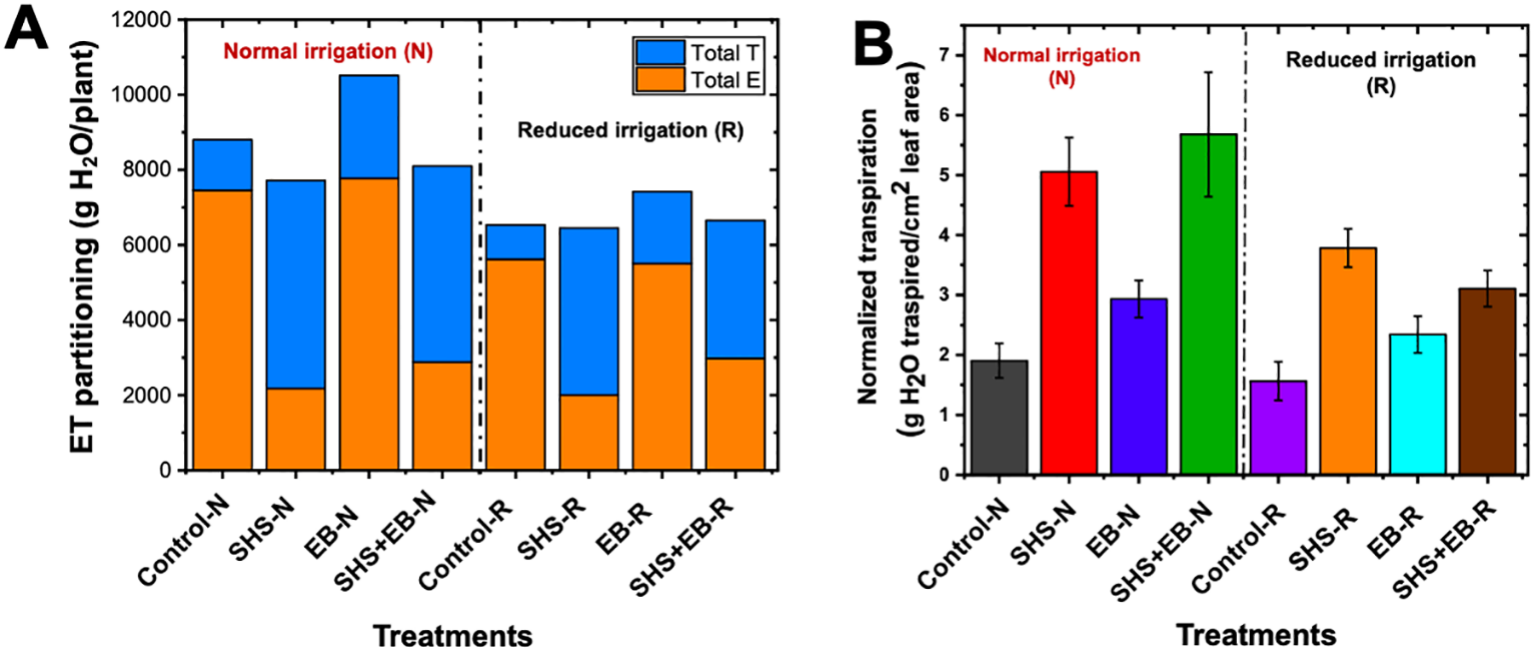
Evapotranspiration (ET) partitioning in *Moringa oleifera* plants. E and T are total evaporation and transpiration per plant, respectively **(A)** for control soil, Superhydrophobic sand (SHS), engineered biochar (EB) and their combination (SHS+EB) under normal (N) and reduced irrigation (R). Total transpiration per plant was normalized per unit leaf area (LA) in each treatment (**B**). Each data point is a mean of five replicates (*n* = 5).

Under **N** irrigation, total evaporation significantly reduced in SHS and SHS+EB treatments by 71% and 61%, respectively; and by 64% (*p*< 0.05) and 47% (*p*< 0.05), respectively under R irrigation (**Figure 6A**). However, no significant difference was found in evaporation between EB and control soil under either irrigation regimes. In effect, total transpiration under N irrigation significantly increased by 311% and 288% in SHS and SHS+EB, respectively compared with the controls. Similarly, under R irrigation, cumulative transpiration was higher in SHS and SHS+EB by 385%, and 301%, respectively. Although we found no reduction in evaporative water loss due to biochar treatment, total transpiration was higher than in the controls by 103% and 110% under N and R irrigation, respectively (*p<* 0.05). When we normalized transpiration values against total leaf area per plant (**Figure 6B**), normalized transpiration values were highest in SHS and SHS+EB both under **N** (166% and 198%, respectively) and R irrigation (142% and 99%, respectively) than in the EB (50–54%) and control treatments.

### 3.4 Leaf chlorophyll content and stomatal conductance

**Figure 7:**
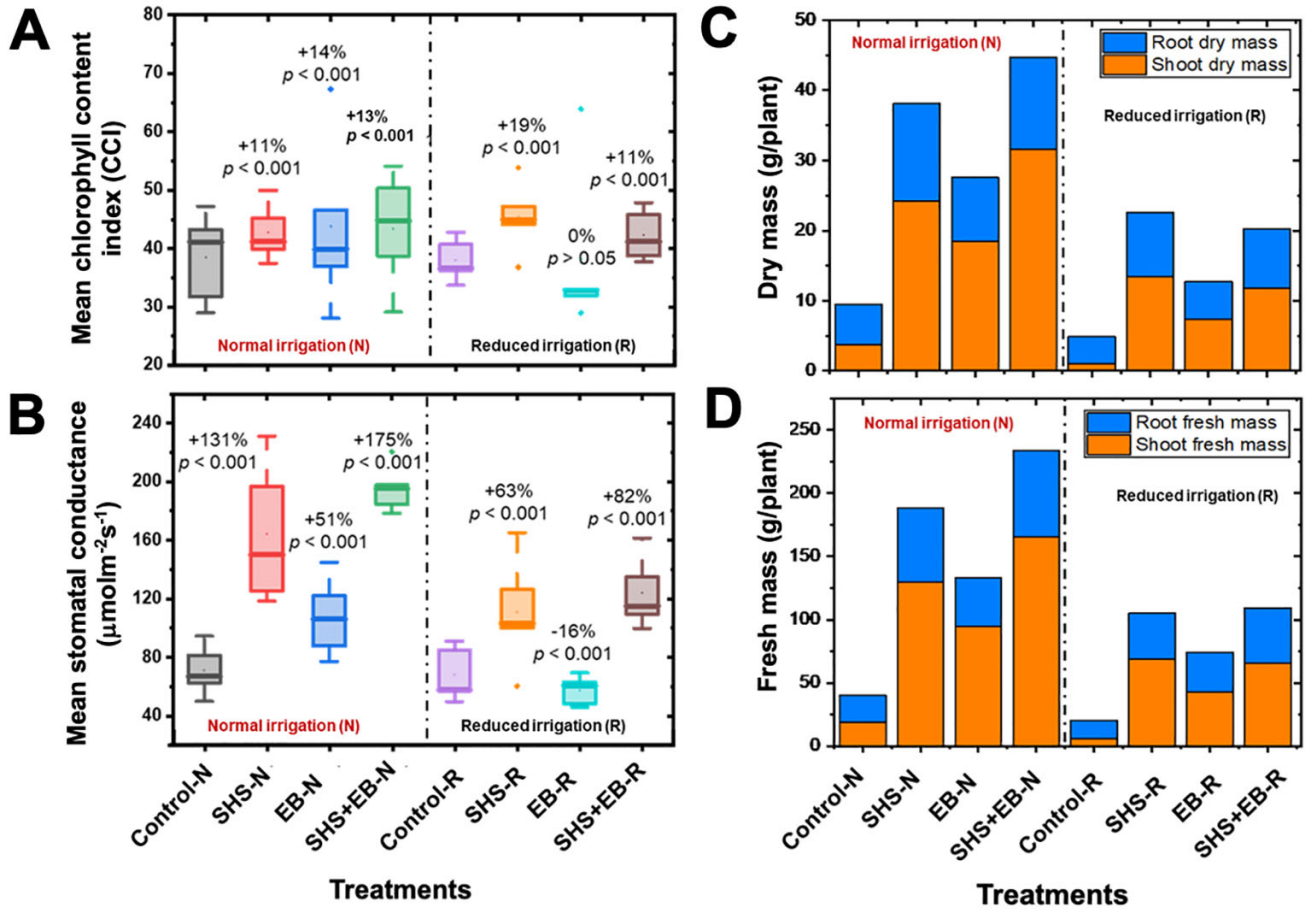
Boxplots showing **(A)** mean leaf chlorophyll content index (CCI); **(B)** mean stomatal conductance for plants grown in control soil, Superhydrophobic sand (SHS), engineered biochar (EB) and their combination (SHS+EB) under normal (N) and reduced (R) irrigation scenarios. **(C)** shoot and root dry mass partitioning; **(D)** shoot and root fresh mass partitioning in different treatments under N and R irrigation. Each box/bar represents the data from 5 samples (*n* = 5). For the box plot, the mid-line indicates the median value, the dot inside the box represents the mean value, the upper and lower sections of the box represent the 25% and 75% of data points, respectively, and the whiskers on the box represent the 1.5 interquartile range, the dot outside the box indicates outlier. Percentage differences between treatments are presented (for box plots) relative to control along with their corresponding *p*-values derived from two-way ANOVA using *p*< 0.05 level of statistical significance.

Our results show that, relative to control soils, total CCI significantly increased in SHS, EB, and SHS+EB by 11%, 14%, and 13%, respectively under N irrigation; and by 19% and 11% under R irrigation for SHS and SHS+EB treatments, respectively (**Figure 7A**). However, we did not find significant increase in CCI in EB under N or R irrigation (*p* > 0.05). Under N irrigation, leaf stomatal conductance increased in SHS, EB and SHS+EB by 131%, 51%, and 175% (*p*< 0.001), respectively relative to the control soils (**Figure 7B**). Under R irrigation, however, EB treatment showed significantly lower stomatal conductance (16%) than the control, whereas conductance in SHS and SHS+EB significantly increased by 63% and 82%, respectively.

### 3.5 Biomass partitioning

All soil amendment treatments had profound effects on both dry and fresh shoot biomass increases than on root biomass (**Figures 7C, D**). Under N irrigation, shoot dry mass increased due to SHS, EB and SHS+EB treatments by 542%, 390%, and 738%, respectively in comparison with the control. Meanwhile, shoot dry mass increased under R irrigation by 1271%, 648%, and 1102% in SHS, EB and SHS+EB treatments, respectively (*p<* 0.005). For roots, dry mass increased under N irrigation due to SHS, EB and SHS+EB by 142%, 52% and 129%, respectively (*p<* 0.05); also, root dry mass increased under R irrigation in SHS, EB and SHS+EB treatments by 133%, 37%, and 115%, respectively (*p<* 0.05).

### 3.6 Metabolomics analysis

**Figure 8:**
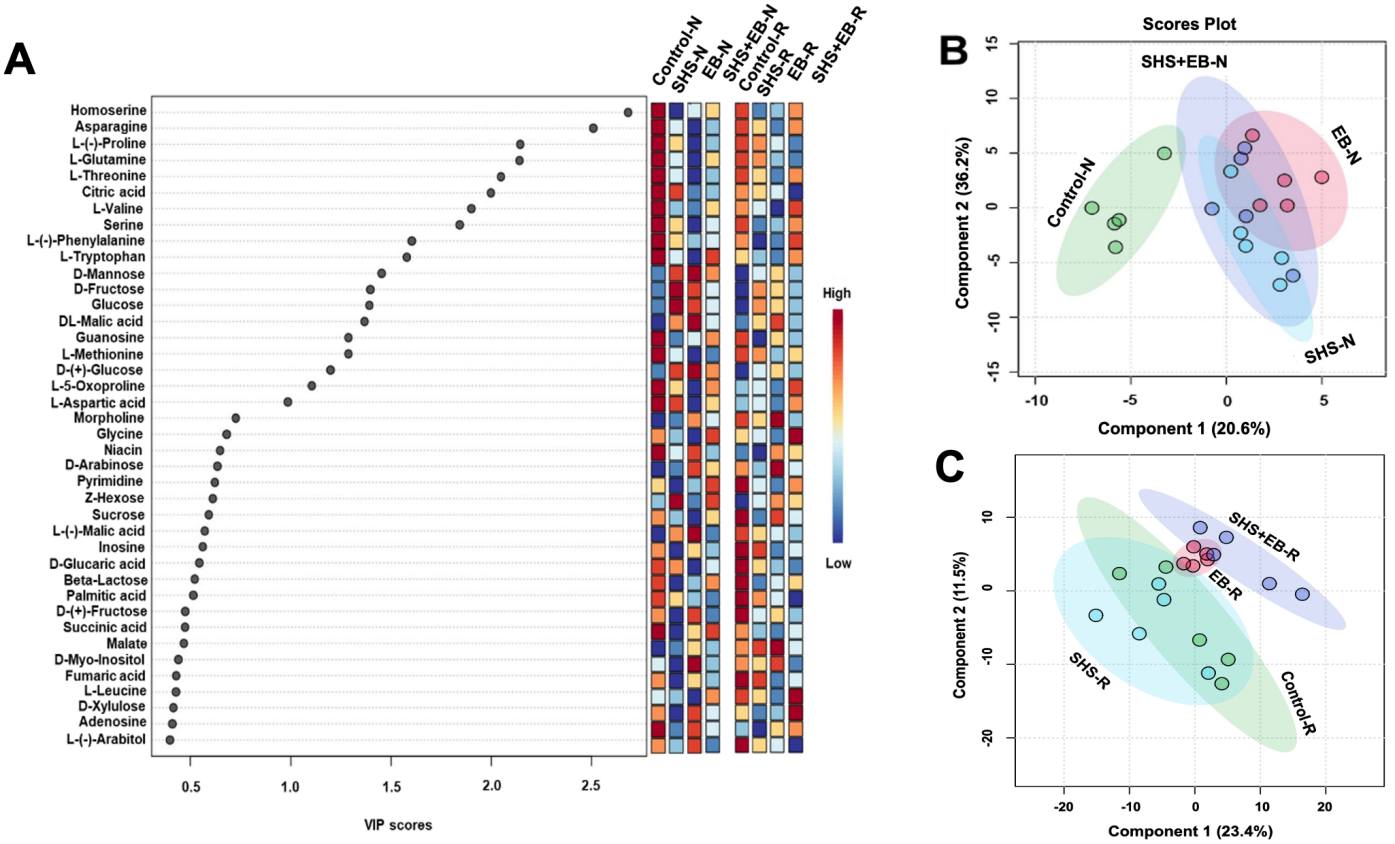
Variable Importance in Projection (VIP) scores for the top 40 metabolites in *Moringa oleifera* plants contributing to variation in metabolic profiles of controls, Superhydrophobic sand (SHS), engineered biochar (EB) and their combination (SHS+EB) under normal (N) and reduced (R) irrigation **(A)**. The colored scale indicates the relative abundance of metabolites ranging from RED to BLUE representing the low and high, respectively. Partial least Square-discriminant analysis (PLS-DA) scores plots of significant metabolites in ANOVA derived for SHS, EB, SHS+EB vs the controls under N irrigation **(B)** and R irrigation **(C)**.

Metabolomic profiling of *M. oleifera* showed significant variation in metabolite accumulation for plants in the control versus those in SHS, EB, and SHS+EB under N and R irrigation (**Figure 8**). In **Figure 8A**, we present the top 40 metabolites contributing to the variation of metabolite profiles in the different treatment groups based on the Variable Importance in Projection (VIP) scores. In this case, we considered that metabolites with a VIP score of ≥ 1.0 are of significance in contributing to the differences between treatment groups (i.e., 19 metabolites involved ranging from Homoserine to L-Aspartic acid). From the colored scale (red to blue), our results demonstrate that plants in the control treatment had the highest concentration or abundance of 13 amino acids (i.e., from Homoserine to L-Tryptophan, L-Methionine, L-5-Oxoproline, L-Aspartic acid) and the nucleoside, guanosine. The abundance of these metabolites apparently followed a similar pattern (i.e., control > SHS > SHS+EB > EB) under both N and R irrigation. For VIP scores > 1.0, SHS and EB had the highest relative abundance of 3 sugars (i.e., D-Mannose, D-Fructose, glucose) and one organic acid (i.e., malic acid) under both irrigation regimes. At VIP scores< 1.0, the relative abundance of sugars (such as sucrose to L-(-)-Arabitol)), fatty acid (palmitic acid), organic acid (succinic acid), and nucleoside (adenosine) was generally higher for plants in control and EB treatments than SHS and SHS+EB treatments under both N and R irrigation. Further analysis using partial least square discriminant analysis (PLS-DA) to provide insights into the metabolic profiles of the plants under different treatments and irrigation rendered a two-component model (**Figures 8B, C**) as calculated by cross-validation (CV). The PLS-DA scores discriminated the control treatment from SHS, EB, and SHS+EB samples into four obvious clusters separated by scores of the first component; the metabolite profile of control samples mostly positioned towards the negative end of component 1 while those of SHS, EB and SHS+EB are mainly positioned at the positive side of component 1 under both irrigation regimes, except SHS under R irrigation. The complete metabolomics and PLSDA VIP Score data can be found in the supplementary data sheets (**Supplementary Data Sheets 1A**, **B**).

### 3.7 Soil physicochemical properties

Soil analysis revealed that pH of the control and SHS-treated soils did not differ before and at the end of the experiment (**Table 1**). When compared with the control soil, the EC and salinity of the soil was lower in SHS+EB treatment by 17 times (94%) and 12 times (92%) under N and R irrigation, respectively. These synergistic effects on soil EC and salinity were higher than the stand-alone effect of SHS or EB, which was about 4–2 times (72–64%) that of the control soil under N and R irrigation. While the concentration of K did not differ between SHS and control soils, EB and SHS+EB-treated soils had 2–4 times less K concentration under N irrigation. Under R irrigation, the concentrations of K in SHS and EB were higher than in the control soils by 19% and 26%, respectively, while SHS+EB-soil did not significantly differ from the control soil. The concentration of P in both SHS and EB treated soils was twice that in the control soil under N irrigation. Whereas P concentration did not differ between SHS and EB treatment under N irrigation, its concentration in SHS+EB treated soil was 19% less than in SHS and EB treatments. Under R irrigation, P concentration in SHS, EB and SHS+EB treated soils was 2–6 times higher than in the control soil (p< 0.05). On the other hand, under N irrigation, total nitrogen (NH_4_–N+NO_3_–N) in SHS, EB and SHS+EB-amended soil was less than in the control soil by 2, 3, and 12 times, respectively. Under R irrigation, total nitrogen concentration in SHS, EB, and SHS+EB was 3–6 times less than in the control. From these results, the efficiency of soil nitrogen (NH_4_–N or NO_3_–N) utilization by plants in SHS+EB treatment (86–95%) was significantly higher than for SHS or EB treatment (58–67%) in both irrigation relative to the control case.

## 4 Discussion

The results presented in this study demonstrate the individual and combined effects of SHS and EB treatments when used as soil amendments in response to the challenges of low water and nutrient use-efficiency for plant growth in arid conditions. Due to its extreme water repellency (**Figures 1C, D**), SHS served as a dry diffusion barrier, and significantly reduced the evaporative water loss from the top soil, as evident in previous studies (Mishra et al., 2022; Gallo Jr et al., 2022). The enhanced soil water retention was then harnessed by the plant via the transpiration process (Odokonyero et al., 2022). Higher transpiration due to SHS (**Figures 5** **,** **6**) boosted photosynthesis, which in turn facilitated faster growth of the plants (i.e., height, trunk diameter, leaf area, and dry mass) in the following order: control< EB< SHS< SHS+EB under N or R irrigation (**Figures 3**, **7**). Increase in plant leaf area due to SHS or SHS+EB application was associated with the higher transpiration in those plants than those in the controls, as indicated by the trends in **Supplementary Figure 1** and **Figure 5C**. However, the total leaf area in the control and EB treatments were not significantly different yet, EB plants had a higher transpiration than in the controls. We speculate that higher transpiration observed in EB-treated plants relative to the controls could be due to enhanced lenticular and cuticular transpiration on the stem (Wang et al., 2018), since the trunk diameter in EB was significantly higher than in the controls (**Figure 3C**).

In terms of nutrients, the EB contributed to enhanced nutrient use efficiency in comparison with the controls. These contributions were reflected in higher plant height, trunk diameter, leaf chlorophyll content, stomatal conductance, biomass, and sugar concentrations of plant leaves (**Figures 3**, **7**, **8**). The synergistic effects of both SHS and EB were clearly manifested through a more enhanced plant growth and physiological responses in SHS+EB compared to the individual effect of each technology. These findings suggest that water availability due to SHS played a greater role in determining plant responses in SHS treatment versus EB, while nutrients played a bigger role in EB versus control plants’ responses. By boosting transpiration, photosynthesis and plant morphological traits (Román-Dañobeytia et al., 2021; Román-Dañobeytia et al., 2015; Patanè, 2011; Fagbenro et al., 2015), both SHS and EB can significantly alleviate water and nutrient stresses, and benefit plant survival in water limited and poor soil conditions. Positive effects of mulches and biochar in *M. oleifera* tree plants have been demonstrated in other contexts; for instance, plastic mulching significantly improved flowering and panicle number in *M. oleifera* plants (Pugalendhi and Pushpanathan, 2018); while *Gliricidia* biochar improved plant height, stem diameter, dry matter yield and root: shoot ratio in moringa (Fagbenro et al., 2015). Consistent with the present study, previous research also indicated that soil water and nutrient availability due to mulches and biochar positively correlated with leaf area, stomatal conductance, transpiration, and biomass (Odokonyero et al., 2022; Patanè, 2011).

For both EB and SHS+EB treatments, the pre-loading of nutrients onto the date palm biochar provided sufficient nutrients for plants at an early stage, which enabled them to outperform traditional NPK fertilizer supplementation in their non-EB treated counterparts (**Figures 3**, **7**). This was surprising even though the total amount of the nutrients in the biochar-based-treatments was lower than that in the cumulative fertilizer added in the rest of the treatments (**Table 1**). We also suspect that our experimental design with sealed pots might have been vulnerable to nutrient accumulation over time, potentially impacting plant growth. This is plausible due to evidence from a previous study showing that *M. oleifera* trees treated with lower amount of nutrients (i.e., 100 kg N ha^−1^ and 80 kg P ha^−1^) performed significantly better than those supplied with higher nutrient levels (i.e., 400 kg N ha^−1^ and 120 kg P ha^−1^) in terms of plant height and stem diameter (Sokombela et al., 2022). SHS suffered less stress than the controls because of higher moisture content in the soil, which reduced the osmotic stress (Peguero-Pina et al., 2020; Yancey et al., 1982). To confirm this hypothesis, our future pot studies will allow for percolation; also, we will apply the EB-treatment without nutrient pre-loading that will receive regular fertilizer supplementation. This will also help ascertain the effects of EB on preventing nutrient leaching.

In **Table 1**, the effect of EB treatment on soil EC, salinity and nutrient concentrations can be seen to vary across both irrigation regimes. The most consistent effect was demonstrated by the SHS+EB treatment combination, where the EC and salinity were the lowest, and the soil macronutrients were significantly depleted to very low concentrations (with the exception of P concentration under R irrigation) compared with SHS and EB treatments. The EB and SHS+EB soils displayed the lowest total nitrogen levels at the end of the experiment, indicating a more efficient uptake of nutrients when compared to the control and SHS treatments. This is corroborated by the higher biomass in EB and SHS+EB-treated plants than in control treatment (**Figures 7C, D**), which indicates that most of the nutrients in the soil were taken up and assimilated by plants to produce carbohydrates, amino acids and other metabolic products (Lawlor, 2002) for plant growth and biomass development. For P and K, the trends were less clear. According to the efficiency of uptake, the residual concentration of K in the soil followed a similar trend as total nitrogen under N irrigation (i.e., control > SHS > EB > SHS+EB), but it was not the case under R irrigation, highlighting the importance of soil moisture for the efficient uptake of nutrients. Other nutrients such as Na and Zn increased slightly in the soil at the end of the experiment, which can be attributed to their accumulation from the irrigation water applied in the absence of percolation due to sealing of the holes at the bottom of the pots.

Following the metabolomics profiling, the high relative abundance of most amino acids, sugars, fatty acid, and organic acid in the leaves of control plants is attributed to the plants’ physiological response to water or nutrient stress (Khan et al., 2020). High evaporative water losses in the control plants (**Figures 6**, **7B**) presented a situation of water stress to the plants. As a result, water stress triggered the biosynthetic pathways for increased production and accumulation of amino acids such as proline, L-Tryptophan, L-Phenylalanine, and Aspartate as shown in **Figure 8A** (Lei et al., 2022; Yasmin et al., 2017; Sheng et al., 2022). These amino acids are essential for maintaining cellular integrity during stress and help in plant recovery from and adaptability to environmental stresses (Hayat et al., 2012; Hare et al., 1998). For instance, proline acts as an osmolyte for osmotic adjustment, and contributes to stabilizing sub-cellular structures (e.g., membranes and proteins), scavenging free radicals and buffering cellular redox potential under stress conditions (Ashraf and Foolad, 2007). Alongside its role in normal development, asparagine also accumulates in response to water stress and nutrient deficiencies (Lea et al., 2007). Despite their exposure to stress, the accumulation of metabolites in the control plants signifies an adaptive strategy for safeguarding them against the severe impact of stress compared with SHS, EB, and SHS+EB plants, which were cushioned against water or nutrient stress. High concentration of non-structural carbohydrates such as D-Mannose, D-Fructose, and glucose in plants grown with SHS or EB treatment under N and R irrigation could be attributed to the enhanced water or nutrient-use efficiency due to increased carbon assimilation resulting into higher rates of photosynthesis than for control plants. This is corroborated by the observed increase in transpiration, stomatal conductance, leaf chlorophyll content, as well as fresh and dry biomass in SHS, EB or SHS+EB treatments (**Figures 6**, **7**).

In closing, considering the material production aspect of this study, it is worth noting that even though the manufacturing of EB required liquified petroleum gas in batch reactors that did not harness the syngas produced in the chamber, continuous biochar reactors present a reliable platform for utilizing the syngas to drive the pyrolysis process. In a pilot or industrial scale set-up, with a typical 5-day run time followed by 2 days for maintenance and rest, the syngas is routed into the outside barrel for combustion, while a conveyer system brings in the feedstock that is pyrolyzed into biochar within minutes. This underscores the carbon sequestration potential and scalability of this approach in pursuit of environmental sustainability.

## 5 Conclusions

The present study investigated the effects of SHS, EB, and their combination on the growth, evapotranspiration partitioning and metabolomics profiling of *M. oleifera* plants under normal and reduced irrigation inside a greenhouse. By suppressing soil evaporation and osmotic stress, SHS significantly enhanced plant transpiration, growth, and biomass production. EB (with pre-loaded nutrients) increased plant growth and biomass even without the need for further fertilizer supplementation for 21 weeks. This demonstrates the potential of EB as an effective fertilizer nutrient carrier that slowly releases nutrients to plants when needed. High accumulation of metabolites (e.g., amino acids, sugars, nucleosides, organic acids and fatty acids) due to amendment application would be fundamental for plant survival under environmental stresses. This study shows that the application of SHS+EB can provide synergistic benefits in terms of improving irrigation water and nutrient-use efficiency in plants. In the Arabian Peninsula and Northeast Africa, *M. peregrina* is cherished for its traditional, nutritional, industrial and medicinal values and its cultivation is desirable due to its resilience and commercial potential. The benefits demonstrated by SHS and EB technologies on *M. oleifera* underscore the potential of these technologies to promote the revegetation of desert ecosystems with such native tree species and mitigate the challenges of water scarcity and soil nutrient deficiency in Saudi Arabia and the Middle East.

## Data Availability

The original contributions presented in the study are included in the article/**Supplementary Material**. Further inquiries can be directed to the corresponding authors.

## Supplementary material

Supplementary Figure 1 Changes in mean leaf area between week 11 and week 15 after transplanting for plants in control treatment, Superhydrophobic sand (SHS), engineered biochar (EB), and their combination (SHS+EB) under normal (N) and reduced (R) irrigation
