# Supplementary material for: Superhydrophobic sand mulch and date palm biochar boost growth of Moringa oleifera in sandy soils via enhanced irrigation and nutrient use efficiency
Source: Front Plant Sci. 2024 Nov 26;15:1434462. doi: 10.3389/fpls.2024.1434462 (PMC11628278; doi:10.3389/fpls.2024.1434462)
Supplement: Supplementary Figure 1 — Changes in mean leaf area between week 11 and week 15 after transplanting for plants in control treatment, Superhydrophobic sand (SHS), engineered biochar (EB), and their combination (SHS+EB) under normal (N) and reduced (R) irrigation [file DataSheet1.pdf]

SUPPLEMENTARY INFORMATION

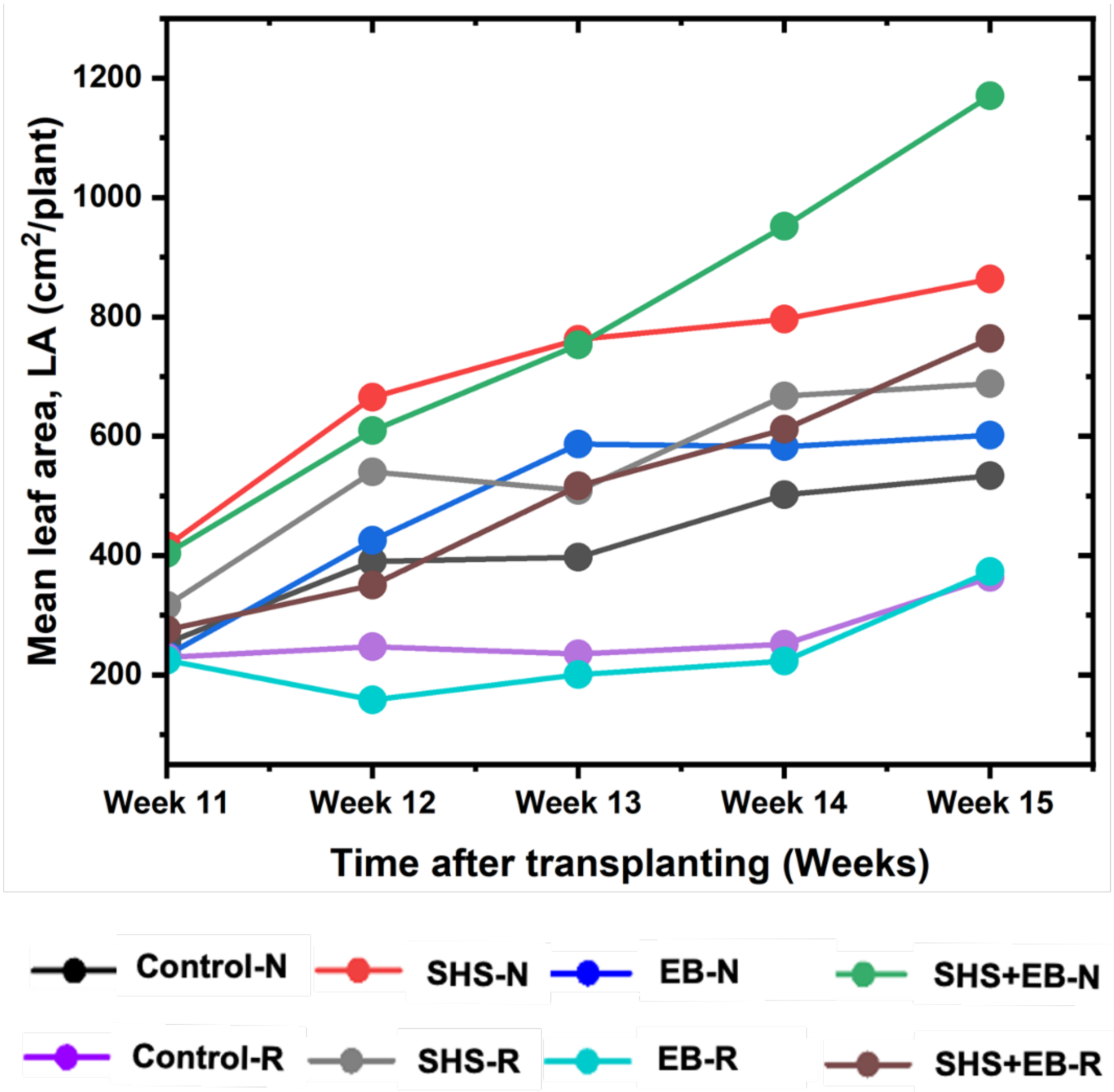

Supplementary Figure, SF1. Changes in mean leaf area between week 11 and week 15 after transplanting for plants in control treatment, Superhydrophobic sand (SHS), engineered biochar (EB), and their combination (SHS+EB) under normal (**N**) and reduced (**R**) irrigation
